# Supplementary material for: Reduced Potential Barrier of Sodium-Substituted Disordered Rocksalt Cathode for Oxygen Evolution Electrocatalysts
Source: Nanomaterials (Basel). 2022 Dec 20;13(1):10. doi: 10.3390/nano13010010 (PMC9824024; doi:10.3390/nano13010010)
Supplement: Supplementary file 1 [file nanomaterials-13-00010-s001.zip › nanomaterials-2111371-supplementary.pdf]

Supplementary Information for

Reduced Potential Barrier of Sodium-substituted Disordered Rocksalt Cathode for Oxygen Evolution Electrocatalysts

Aditya Narayan Singh[1], Amir Hajibabaei[2], Miran Ha[2], Abhishek Meena[3], Hyun-Seok Kim[4], Chinna Bathula[4*], Kyung-Wan Nam[1, 5,*]

[1]Department of Energy and Materials Engineering, Dongguk University-Seoul, Seoul 04620, Republic of Korea
[2]Center for Superfunctional Materials, Department of Chemistry, Ulsan National Institute of Science and Technology (UNIST), 50, UNIST-gil, Ulsan 44919, Republic of Korea
[3]Division of Physics and Semiconductor Science, Dongguk University-Seoul, Seoul 04620, Republic of Korea
[4]Division of Electronics and Electrical Engineering, Dongguk University–Seoul, Seoul 04620, Republic of Korea
[5]Center for Next Generation Energy and Electronic Materials, Dongguk University-Seoul, Seoul 04620, Republic of Korea
* Correspondence: cdbathula@dongguk.edu (C.D.); knam@dongguk.edu (K.-W.N.)

Table S1. The ICP-OES results of synthesized powders. The data shows the number of atoms per mole of material. The ICP-OES data were taken several times and the best data obtained are presented here with a round off to the nearest two decimal places. The data in parentheses () denotes the theoretical values.

Samples	Li	Ru	Ni	Na
**1**	1.22 (1.23)	0.61(0.61)	0.16(0.16)	/
**2**	1.22 (1.23)	0.61(0.61)	0.10(0.11)	0.05(0.05)

Table S2. A comparative chart for overpotential (η) of cathode materials used as OER electrocatalyst.

Catalysts	η @ 10 mA cm^{-2} (mV)	Condition (Electrolyte)	Refs.
**2**	270	1.0M KOH	this work
**1**	296	1.0M KOH	this work
$LiCo_{0.8}Fe_{0.2}O_2$	340	0.1M KOH	[1]
$LiCoO_2$	430	0.1M KOH	[1]
$LiCo_{0.33}Ni_{0.33}Fe_{0.33}O_2$	420	0.1M KOH	[2]
$LiNi_{0.8}Fe_{0.2}O_2$	302	0.1M KOH	[3]
$Li_{0.7}Co_{0.75}Fe_{0.25}PO_4$	390	6.0M KOH	[4]
$Ni_{30}Fe_7Co_{20}Ce_{43}O_x$	370	1.0M KOH	[5]
$Ni_{0.9}Fe_{0.1}O_x$	340	1.0M KOH	[6]
NiCo LDH NSs	370	1.0M KOH	[7]
$Co_2V_2O_7$/VN	320	1.0M KOH	[8]
c-$CoMn_2$/C	560	0.1M KOH	[9]
NiCo LDH NSs/CP	370	1.0M KOH	[7]
$NiCo_2O_4$ ultrathin NSs	320	1.0M KOH	[10]
Ni-doped NW Co_3O_4/TF	370	1.0M NaOH	[11]
$Ba_{0.5}Sr_{0.5}Co_{0.8}Fe_{0.2}O_{3-\delta}$	350	0.1M KOH	[12]
$Ni_{0.75}V_{0.25}$-LDH	320	1.0M KOH	[13]
$NiCo_2O_3$@OMC	281	1.0M KOH	[14]
Amorphous $CoFeO_x$	490	0.1M KOH	[15]
$CoV_{1.5}Fe_{0.5}O_4$	300	0.1M KOH	[16]
Ni/Mo_2C-NCNFs	288	1.0M KOH	[17]
$(Pr_{0.5}Ba_{0.5})CoO_{3-\delta}$	350	0.1M KOH	[18]
V-doped NiS_2	290	1.0M KOH	[19]
RuO_2	398	1.0M KOH	[20]
IrO_2	343	1.0M KOH	[21]

Supplementary discussions:

S1. Effect of Na-doping on binding energy

Chemical doping of the elements in their trace amounts is a followed trend to obtain tailor-made electrocatalysts for water splitting.[22] To highlight the impact of Na-doping in **2**, their core-level spectra have been explained in **Figure 1b-f.** Few other essential aspects will be discussed here. Doping with Na results in surplus oxygen which can catalyze the OER. As the surface oxygen plays a significant role in determining the reaction kinetics in OER, the availability of some of the loosely bound surface oxygen's in **2** (5.45 eV) compared to **1** (6.51 eV) accentuates the facile diffusion of O_2 gas consistent with the experiment data (**Figure 3b and S3**). The XPS data from oxygen 1s core-level spectra for carbonate/hydroxides/oxyhydroxides species (CO_3^{2-}/OH^-/OOH: BE ~531.48 eV) in **2** is much lower than **1**(~531.7 eV), further supports that surface oxygen in **2** are loosely bound and can quickly diffuse during the reaction. Remember here that these species (OH^-/OOH*, where * denotes the active sites of catalyst) are the absorbed intermediate and rate-determining species in OER.[23] The presence of these intermediates in **2** further supports better reaction kinetics against **1**.

S2. Structural characterizations

As the morphology of the particle largely influences the activity of several electrocatalytic reactions, including OER,[24] the detailed analysis of their shape becomes vital. As can be seen in **Figure 2a**, the particles in **2** have faceted structures. These faceted structures are believed to facilitate better reaction surface for accommodating the reaction intermediate[25], thus improving the overall reaction kinetics. These faceted structures can be seen decorated by small-sized particles, which are beneficial in providing better connectivity between particle-to-particle and improving the electrical conductivity.[26] Besides, these decorations also make the surface rough and make percolation of electrolyte through a larger exposed surface, thereby increasing OER, which are predominantly surface-dependent reactions. On the contrary, no such features are available in **1** (**Figure S2**).

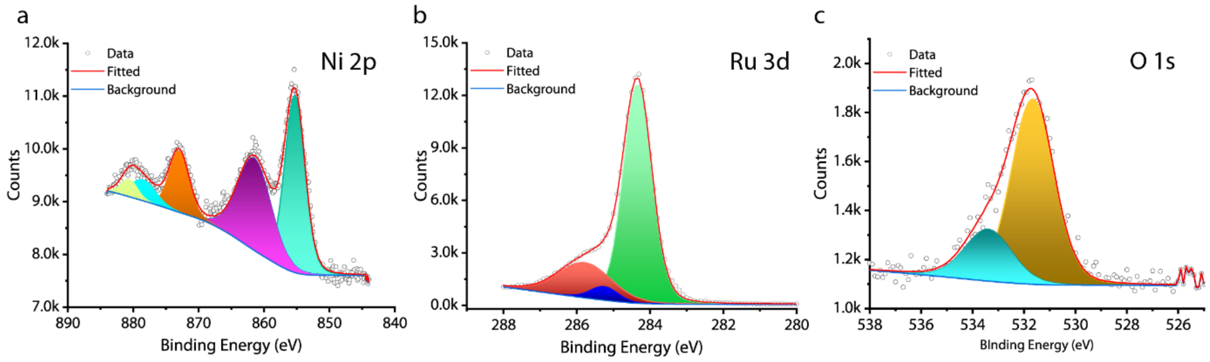

Figure S1. Core-level XPS spectra of **1**. (a) Ni 2p. (b) Ru 3d. (c) O 1s.

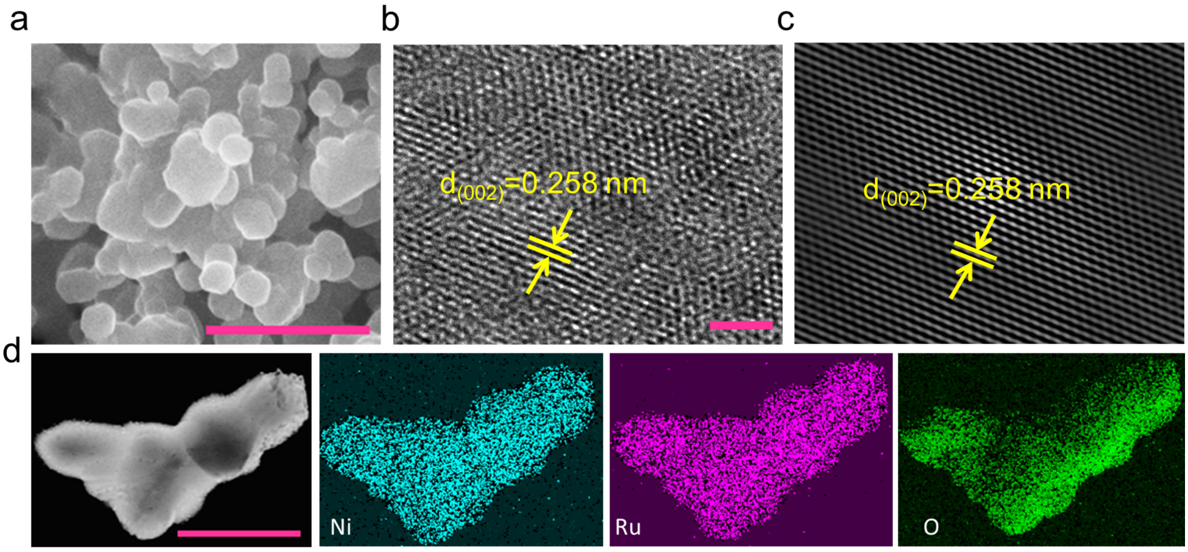

Figure S2. Structural and elemental characterizations of **1**. (a) SEM image (scale bar 2 μm). (b) HRTEM image showing lattice fringe distance 0.258 nm corresponding to (002). (scale bar 2 nm). (c) the autocorrelated image corresponding to b. (d) High-angle annular dark-field scanning transmission electron microscopy (HAADF-STEM) and EDS elemental mapping of **1** (scale bar 250 nm).

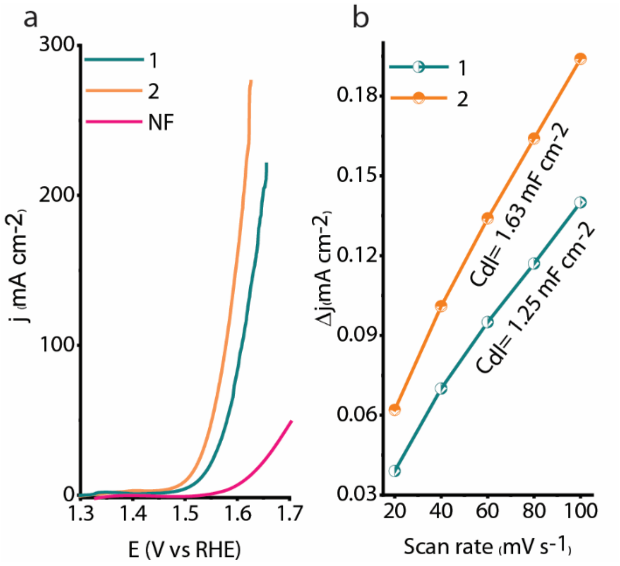

Figure S3. Comparative LSV curves of **1**, **2**, and NF. b) Plots of charging current density differences (Δj) vs. the scan rate for electrodes.

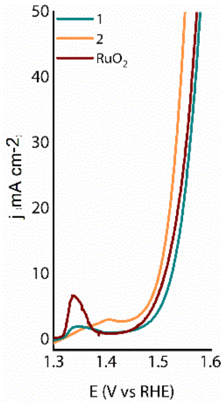

Figure S4. Comparative LSV curves of **1**, **2**, and RuO_2.

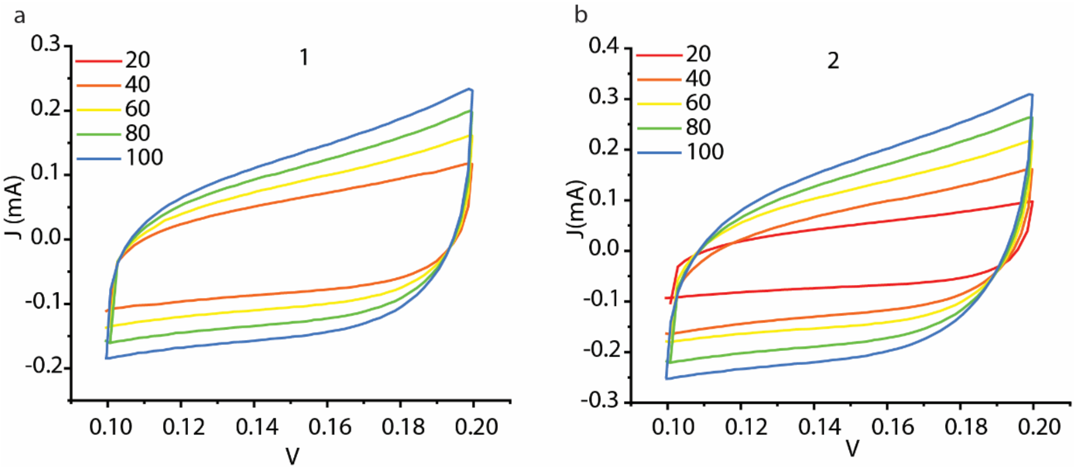

Figure S5. Cyclic voltammograms compared at different scan rate (a) **1**.(b) **2**.

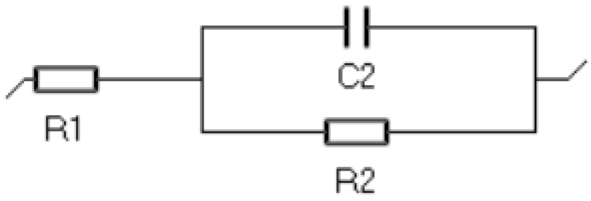

Figure S6. Equivalent circuit.

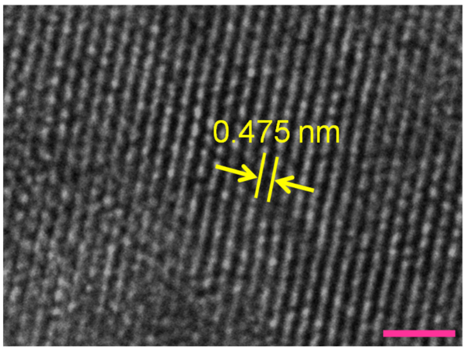

Figure S7. HRTEM image after long-term stability (scale bar 2 nm). The lattice fringe distance after long-term stability remains almost same to that of Figure 2b.

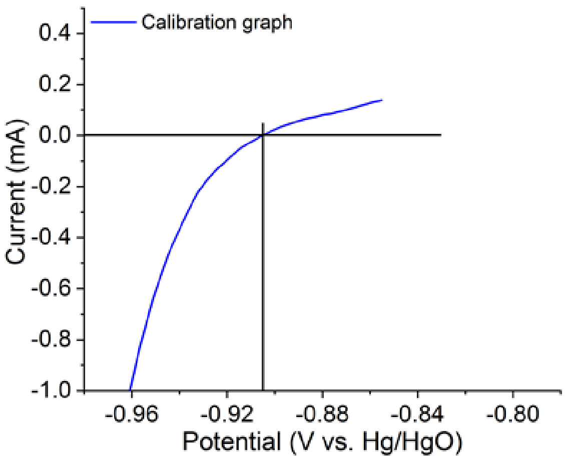

Figure S8. Calibration of the Hg/HgO reference electrode. For all measurements, Hg/HgO was used as the reference electrode. The Hg/HgO calibration with respect to the reversible hydrogen electrode (RHE) was performed in the high purity H_2 saturated electrolyte with Pt wire as the working electrode. LSV was run at a rate of 1 mV s^{-1}, and potential at zero mA current was taken to be the thermodynamic potential for hydrogen evolution reactions. So, in 1M KOH, E(RHE) = E(Hg/HgO) + 0.905

References:

1 Zhu, Y.; Zhou, W.; Chen, Y.; Yu, J.; Liu, M. Shao, Z. A High-Performance Electrocatalyst for Oxygen Evolution Reaction: LiCo0.8Fe0.2O2. *Adv. Mater.* **27**, 7150-7155 (2015). https://doi.org:10.1002/adma.201503532

2 Lu, Z. *et al.* Electrochemical tuning of layered lithium transition metal oxides for improvement of oxygen evolution reaction. *Nat. Commun.* **5**, 4345 (2014). https://doi.org:10.1038/ncomms5345

3 Zhu, K.; Wu, T.; Zhu, Y.; Li, X.; Li, M.; Lu, R.; Wang, J.; Zhu, X. Yang, W. Layered Fe-Substituted LiNiO2 Electrocatalysts for High-Efficiency Oxygen Evolution Reaction. *ACS Energy Lett.* **2**, 1654-1660 (2017). https://doi.org:10.1021/acsenergylett.7b00434

4 Gershinsky, Y. Zitoun, D. Direct Chemical Synthesis of Lithium Sub-Stochiometric Olivine Li0. 7Co0. 75Fe0. 25PO4 Coated with Reduced Graphene Oxide as Oxygen Evolution Reaction Electrocatalyst. *ACS Catal.* **8**, 8715-8725 (2018).

5 Haber, J. A.; Cai, Y.; Jung, S.; Xiang, C.; Mitrovic, S.; Jin, J.; Bell, A. T. Gregoire, J. M. Discovering Ce-rich oxygen evolution catalysts, from high throughput screening to water electrolysis. *Energ. Environ. Sci.* **7**, 682-688 (2014).

6 Trotochaud, L.; Ranney, J. K.; Williams, K. N. Boettcher, S. W. Solution-cast metal oxide thin film electrocatalysts for oxygen evolution. *J. Am. Chem. Soc.* **134**, 17253-17261 (2012).

7 Liang, H.; Meng, F.; Cabán-Acevedo, M.; Li, L.; Forticaux, A.; Xiu, L.; Wang, Z. Jin, S. Hydrothermal continuous flow synthesis and exfoliation of NiCo layered double hydroxide nanosheets for enhanced oxygen evolution catalysis. *Nano Lett.* **15**, 1421-1427 (2015).

8 Peng, X. *et al.* In situ segregation of cobalt nanoparticles on VN nanosheets via nitriding of Co2V2O7 nanosheets as efficient oxygen evolution reaction electrocatalysts. *Nano Energy* **34**, 1-7 (2017). https://doi.org:https://doi.org/10.1016/j.nanoen.2017.02.016

9 Li, C.; Han, X.; Cheng, F.; Hu, Y.; Chen, C. Chen, J. Phase and composition controllable synthesis of cobalt manganese spinel nanoparticles towards efficient oxygen electrocatalysis. *Nat. Commun.* **6**, 7345 (2015). https://doi.org:10.1038/ncomms8345

10 Bao, J. *et al.* Ultrathin spinel-structured nanosheets rich in oxygen deficiencies for enhanced electrocatalytic water oxidation. *Angew. Chem.* **127**, 7507-7512 (2015).

11 Lu, Z.; Xu, W.; Zhu, W.; Yang, Q.; Lei, X.; Liu, J.; Li, Y.; Sun, X. Duan, X. Three-dimensional NiFe layered double hydroxide film for high-efficiency oxygen evolution reaction. *Chem. Commun.* **50**, 6479-6482 (2014).

12 Suntivich, J.; May, K. J.; Gasteiger, H. A.; Goodenough, J. B. Shao-Horn, Y. A perovskite oxide optimized for oxygen evolution catalysis from molecular orbital principles. *Science* **334**, 1383-1385 (2011).

13 Fan, K. *et al.* Nickel–vanadium monolayer double hydroxide for efficient electrochemical water oxidation. *Nat. Commun.* **7**, 1-9 (2016).

14 Zhang, Y.; Wang, X.; Luo, F.; Tan, Y.; Zeng, L.; Fang, B. Liu, A. Rock salt type NiCo2O3 supported on ordered mesoporous carbon as a highly efficient electrocatalyst for oxygen evolution reaction. *Appl. Catal., B* **256**, 117852 (2019). https://doi.org:https://doi.org/10.1016/j.apcatb.2019.117852

15 Indra, A.; Menezes, P. W.; Sahraie, N. R.; Bergmann, A.; Das, C.; Tallarida, M.; Schmeißer, D.; Strasser, P. Driess, M. Unification of catalytic water oxidation and oxygen reduction reactions: amorphous beat crystalline cobalt iron oxides. *J. Am. Chem. Soc.* **136**, 17530-17536 (2014).

16 Chakrapani, K. *et al.* The Role of Composition of Uniform and Highly Dispersed Cobalt Vanadium Iron Spinel Nanocrystals for Oxygen Electrocatalysis. *ACS Catal.* **8**, 1259-1267 (2018). https://doi.org:10.1021/acscatal.7b03529

17 Li, M.; Zhu, Y.; Wang, H.; Wang, C.; Pinna, N. Lu, X. Ni Strongly Coupled with Mo2C Encapsulated in Nitrogen-Doped Carbon Nanofibers as Robust Bifunctional Catalyst for Overall Water Splitting. *Adv. Energy Mater.* **9**, 1803185 (2019). https://doi.org:10.1002/aenm.201803185

18 Grimaud, A.; May, K. J.; Carlton, C. E.; Lee, Y.-L.; Risch, M.; Hong, W. T.; Zhou, J. Shao-Horn, Y. Double perovskites as a family of highly active catalysts for oxygen evolution in alkaline solution. *Nat. Commun.* **4**, 2439 (2013). https://doi.org:10.1038/ncomms3439

19 Liu, H.; He, Q.; Jiang, H.; Lin, Y.; Zhang, Y.; Habib, M.; Chen, S. Song, L. Electronic structure reconfiguration toward pyrite NiS2 via engineered heteroatom defect boosting overall water splitting. *ACS nano* **11**, 11574-11583 (2017).

20 Fan, K. *et al.* Direct Observation of Structural Evolution of Metal Chalcogenide in Electrocatalytic Water Oxidation. *ACS Nano* **12**, 12369-12379 (2018). https://doi.org:10.1021/acsnano.8b06312

21 Tiwari, J. N.; Dang, N. K.; Sultan, S.; Thangavel, P.; Jeong, H. Y. Kim, K. S. Multi-heteroatom-doped carbon from waste-yeast biomass for sustained water splitting. *Nat. Sustain.* **3**, 556-563 (2020). https://doi.org:10.1038/s41893-020-0509-6

22 Qu, M. *et al.* Tailoring the Electronic Structures of the La2NiMnO6 Double Perovskite as Efficient Bifunctional Oxygen Electrocatalysis. *Chem. Mater.* **33**, 2062-2071 (2021). https://doi.org:10.1021/acs.chemmater.0c04527

23 Sultan, S.; Tiwari, J. N.; Singh, A. N.; Zhumagali, S.; Ha, M.; Myung, C. W.; Thangavel, P. Kim, K. S. Single Atoms and Clusters Based Nanomaterials for Hydrogen Evolution, Oxygen Evolution Reactions, and Full Water Splitting. *Adv. Energy Mater.* **0**, 1900624 (2019). https://doi.org:10.1002/aenm.201900624

24 Baumung, M.; Schönewald, F.; Erichsen, T.; Volkert, C. A. Risch, M. Influence of particle size on the apparent electrocatalytic activity of LiMn2O4 for oxygen evolution. *Sustain. Energy Fuels* **3**, 2218-2226 (2019). https://doi.org:10.1039/C8SE00551F

25 Wang, H. *et al.* Self-selective catalyst synthesis for CO2 reduction. *Joule* **3**, 1927-1936 (2019).

26 Tian, Z.; Wang, J.; Liu, S.; Li, Q.; Zeng, G.; Yang, Y. Cui, Y. Na-stabilized Ru-based lithium rich layered oxides with enhanced electrochemical performance for lithium ion batteries. *Electrochim. Acta* **253**, 31-38 (2017). https://doi.org:https://doi.org/10.1016/j.electacta.2017.09.032
